# Paralysis of the Hand and Wrist Cured by Strychnine

**Published:** 1844-05-04

**Authors:** 


### PARALYSIS OF THE HAND AND WRIST CURED BY STRYCHNINE.

J. W., ætat. 29, applied as an out-patient of the Royal Free Infirmary on the 20th of June. She had suffered from severe headach for many weeks, accompanied with tinnitus aurium, and great sense of lassitude. On the 19th (the preceding day) she suddenly, whilst engaged in some trifling act, lost all control over the wrist-joint, hand, and fingers. There were no apparent indications of increased arterial excitement, except a slight jerking of the pulse.

Mr. Gay directed her to lose twelve ounces of blood by cupping from the back of the neck, and to be purged with the saline aperient mixture, and a calomel pill of three grains at bedtime.

23. Head slightly relieved; bowels opened; less jerk in the pulse, but no improvement in the extremity. A blister to the back of the neck, to be dressed with savine cerate. Bitter infusion and aperient mixture, equal parts; a wineglassful three times a day.

29. Head much better, very slight uneasiness remaining; appetite good; bowels regular; hand quite useless.

July 11. She has continued her medicines; kept blister open; bathed the extermity daily in warm salt and water, and followed the bath by friction, but no relief to the palsy has taken place. Strychnine, one-twelfth of a grain, three times a day. Blister allowed to heal.

18. She reported that after taking the first pill she suffered from convulsive movements of various parts of the body, accompanied with partial unconsciousness. These symptoms continued for about three hours, and returned, but with gradually diminished violence, after each dose of the strychnine. The effect, however, in the paralysed hand, was very marked, for she began to regain her usual control over it after the first pill, and *this* led her to continue to take the medicine, although its effects appeared to be of so formidable a nature.

Aug. 4. The use of the head completely restored.

Several cases have lately presented themselves at the Royal Free Hospital, in which, as in the foregoing instance, paralysis (after the exciting cause has been removed by appropriate treatment), whatever that cause might have been, has yielded to the influence which strychnine undoubtedly possesses over the motor nerves.—*Lancet.*

